# Genome mining of 2-phenylethanol biosynthetic genes from *Enterobacter* sp. CGMCC 5087 and heterologous overproduction in *Escherichia coli*

**DOI:** 10.1186/s13068-018-1297-3

**Published:** 2018-11-08

**Authors:** Changqing Liu, Kai Zhang, Wenyan Cao, Ge Zhang, Guoqiang Chen, Haiyan Yang, Qian Wang, Haobao Liu, Mo Xian, Haibo Zhang

**Affiliations:** 1 CAS Key Laboratory of Biobased Materials, Qingdao Institute of Bioenergy and Bioprocess Technology, Chinese Academy of Sciences, No.189 Songling Road, Laoshan District, Qingdao, 266101 China; 2 University of Chinese Academy of Sciences, Beijing, China; 3 Key Laboratory for Tobacco Gene Resources’ Tobacco Research Institute, Chinese Academy of Agricultural Sciences, Qingdao, 266101 People’s Republic of China

**Keywords:** 2-Phenylethanol, *Enterobacter* sp. CGMCC 5087, 2-Keto acid decarboxylase, Alcohol dehydrogenase, Phenylpyruvate pathway, Metabolic engineering

## Abstract

**Background:**

2-Phenylethanol (2-PE) is a higher aromatic alcohol that is widely used in the perfumery, cosmetics, and food industries and is also a potentially valuable next-generation biofuel. In our previous study, a new strain *Enterobacter* sp. CGMCC 5087 was isolated to produce 2-PE from glucose through the phenylpyruvate pathway.

**Results:**

In this study, candidate genes for 2-PE biosynthesis were identified from *Enterobacter* sp. CGMCC 5087 by draft whole-genome sequence, metabolic engineering, and shake flask fermentation. Subsequently, the identified genes encoding the 2-keto acid decarboxylase (Kdc) and alcohol dehydrogenase (Adh) enzymes from *Enterobacter* sp. CGMCC 5087 were introduced into *E. coli* BL21(DE3) to construct a high-efficiency microbial cell factory for 2-PE production using the prokaryotic phenylpyruvate pathway. The enzymes Kdc4427 and Adh4428 from *Enterobacter* sp. CGMCC 5087 showed higher performances than did the corresponding enzymes ARO10 and ADH2 from *Saccharomyces cerevisiae*, respectively. The *E*. *coli* cell factory was further improved by overexpressing two upstream shikimate pathway genes, *aroF/aroG/aroH* and *pheA*, to enhance the metabolic flux of the phenylpyruvate pathway, which resulted in 2-PE production of 260 mg/L. The combined overexpression of *tktA* and *ppsA* increased the precursor supply of erythrose-4-phosphate and phosphoenolpyruvate, which resulted in 2-PE production of 320 mg/L, with a productivity of 13.3 mg/L/h.

**Conclusions:**

The present study achieved the highest titer of de novo 2-PE production of in a recombinant *E. coli* system. This study describes a new, efficient 2-PE producer that lays foundation for the industrial-scale production of 2-PE and its derivatives in the future.

**Electronic supplementary material:**

The online version of this article (10.1186/s13068-018-1297-3) contains supplementary material, which is available to authorized users.

## Background

2-Phenylethanol (2-PE), an aromatic alcohol with a rose-like fragrance, is commonly used as a flavor component in the perfumery, cosmetics, and food industries, and it is also a candidate molecule for next-generation biofuels due to its high energy potential [1]. In addition, 2-PE is an important compound for the production of derivatives such as styrene, phenylethyl acetate, and other valuable compounds [2].

**Fig. 1:**
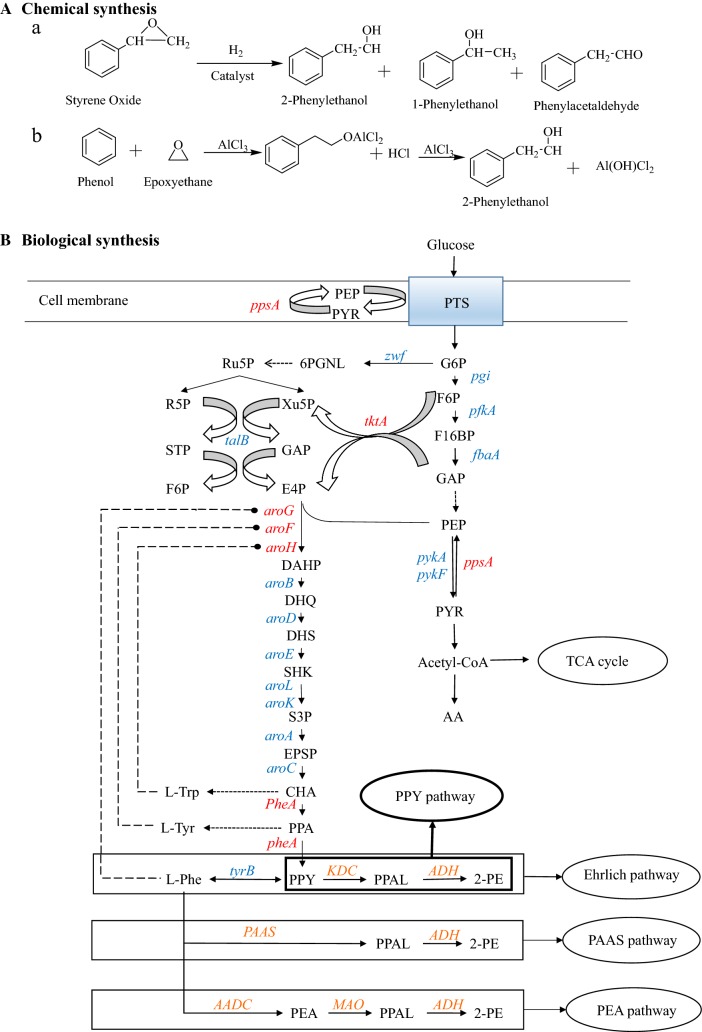
Synthetic route of 2-PE. **A** 2-PE chemical synthesis. **a** Friedel–Craft reaction of ethylene oxide. **b** Catalytic reduction of styrene oxide. **B** 2-PE biosynthesis in *E. coli*. Metabolite abbreviations: PTS, phosphotransferase system; G6P, glucose 6-phosphate; F6P, fructose-6-phosphate; F16BP, fructose-1,6-diphosphate; GAP, glyceraldehyde-3-phosphate; PEP, phosphoenolpyruvate; R5P, ribose-5-phosphate; Xu5P, ribulose-5-phosphate; STP, sedoheptulose-7-phosphate; E4P, erythrose 4-phosphate; DAHP, 3-deoxy-d-arabino-heptulosonate-7-phosphate; DHQ, 3-dehydroquinate; DHS, 3-dehydro-shikimate; SHK, shikimate; S3P, shikimate-3-phosphate; EPSP, 5-enolpyruvylshikimate-3-phosphate; CHA, chorismate; PPA, prephenate; PPY, phenylpyruvate; PPAL, phenylacetaldehyde; PEA, phenylethylamine; l-Phe, l-phenylalanine; l-Tyr, l-tyrosine; l-Trp, l-tryptophan. Genes and enzymes: *zwf*, glucose 6-phosphate dehydrogenase; *pgi*, glucose 6-phosphate isomerase; *fbaA*, fructose-1,6-diphosphate aldolase; *pykA*, pyruvate kinase II; *pykF*, pyruvate kinase I; *ppsA*, phosphoenolpyruvate synthase; *tktA*, transketolase; *talB*, transaldolase B; *aroG*, DAHP synthetase feedback inhibited by Phe; *aroH*, DAHP synthetase feedback inhibited by Trp; *aroF*, DAHP synthetase feedback inhibited by Tyr; *aroB*, 3-dehydroquinate synthase; *aroD*, 3-dehydroquinate dehydratase; *aroE*, shikimate dehydrogenase; *aroL*, shikimate kinase 2; *aroK*, Shikimate kinase 1; *aroA*, 3-phosphoshikimate 1-carboxyvinyltransferase; *aroC*, chorismate synthase; *pheA*, fused chorismate mutase and prephenate dehydratase; *tyrB*, aromatic-amino-acid aminotransferase; *AADC*, aromatic amino acid decarboxylase; *MAO,* amine oxidase; *KDC*, alpha-keto-acid decarboxylase; *Adh*, alcohol dehydrogenase; *PAAS*, phenylacetaldehyde synthase; TCA cycle, tricarboxylic acid cycle

Currently, 2-PE is mainly produced by two chemical processes: (1) styrene oxide reduced with H_2_ to produce mixtures of 2-PE and its derivatives (Fig. 1a) [3] and (2) ethylene and benzene conversion to 2-PE in the presence of molar quantities of aluminum chloride through the Friedel–Craft reaction (Fig. 1b) [4]. In addition, 2-PE is also a byproduct of the production of propylene oxide [2, 5]. Chemical production processes are considered environmentally unfriendly due to their requirements for high temperature, high pressure, and strong acids or alkalis. Furthermore, these processes are connected with the production of unwanted byproducts, thus reducing efficiency and increasing downstream costs [5]. In addition, US and European legislations have restricted the usage of the chemically synthesized 2-PE in some applications, especially in the food industries and cosmetic products [6]. Natural 2-PE is obtained by extraction from the essential oils of plants and flowers. However, this process is costly and inefficient, and cannot satisfy the large market [7]. Therefore, the bioproduction of 2-PE by microorganisms is a promising alternative to the traditional preparation processes.

In nature, there are several ways to synthesize 2-PE, including phenylacetaldehyde synthase (PAAS) pathway, Phenylethylamine (PEA) pathway and the Ehrlich pathway (Fig. 1B) [1, 2, 8–10]. PEA pathway is present in several mammalian tissues and rarely in microorganisms [11]. PAAS pathway mainly exists in plants with a unique dual functionality enzyme PAAS, which could catalyze l-Phe into phenylacetaldehyde directly [10, 12]. Among them, the Ehrlich pathway is thought to be the most significant pathway in eukaryotes. In the Ehrlich pathway, l-Phe is transaminated to phenylpyruvate (PPY) by a transaminase, which is decarboxylated to phenylacetaldehyde (PPAL) by phenylpyruvate decarboxylase, and then reduced to 2-PE by alcohol dehydrogenase [13, 14]. The most prominent microorganisms that carry out the Ehrlich pathway are yeasts, including *Saccharomyces cerevisiae* [15], *Kluyveromyces marxianus* [16], and *Zygosaccharomyces rouxii* [17]. Microbial bioconversion of l-Phe is an effective strategy for producing 2-PE. For instance, Kim et al. reported the use of 10 g/L l-Phe as a sole nitrogen source to produce 4.8 g/L 2-PE in *S. cerevisiae* by overexpressing amino acid transaminases (*ARO9*), phenylpyruvate decarboxylase (*ARO10*) and *Aro80* (a member of the Zn_2_Cys_6_ proteins family, which activates expression of the *ARO9* and *ARO10* genes in response to aromatic amino acids) in an *ALD3* (alcohol dehydrogenase, competing with 2-PE production) deletion strain [13]. In another example, a recombinant *Escherichia coli* harboring a coupled reaction pathway comprising of aromatic transaminase, phenylpyruvate decarboxylase, carbonyl reductase, and glucose dehydrogenase as a catalyst, produced an approximately 96% final product conversion yield of 2-phenylethanol from 40 mM l-Phe [18]. Although the Ehrlich pathway is the main method used for industrial fermentation, the conversion rate from l-Phe to 2-PE is very high, and this process is always faced with an unavoidable problem: the excessively high cost of feedstock l-Phe, which is the main limiting factor for 2-PE production by the Ehrlich pathway.

Thus, de-novo synthesis of 2-PE from glucose via the shikimate pathway is a promising pathway. Erythrose-4-phosphate (E4P) and phosphoenolpyruvate (PEP) from glycolysis and the pentose-phosphate pathway, respectively, are condensed. Subsequently, the intermediates chorismate and prephenate are converted to phenylpyruvate, and then phenylpyruvate reacts through the Ehrlich pathway to synthesize 2-PE. This synthesis process is also called the phenylpyruvate pathway [2]. Compared with the Ehrlich pathway, the phenylpyruvate pathway has a great advantage due to its production of 2-PE at low cost and using renewable sugar as a raw material. Yeasts have been reported to produce 2-PE de novo from glucose; however, the final concentration of 2-PE in culture is very low. For this reason, all current fermentation methods for 2-PE use l-Phe as feedstock. In addition, the yeast fermentation process usually takes several days, which leads to low production of 2-PE [7, 19, 20].

Bacteria, especially *E. coli*, are an attractive host organism because they have unparalleled rapid growth kinetics, simple media requirements, high cell densities, and readily transform DNA [21]. *E. coli* has been successfully engineered to produce a wide range of biofuels and chemicals, including 1-propanol, 1-butanol, 1,4-butanediol, 2,3-butanediol, isopropanol, and (R)-1,2-phenylethanediol [22, 23]. In alcohol production strategies, one critical enzyme is 2-keto-acid decarboxylase (KDC), which is common in plants, yeasts and fungi but less so in bacteria [13–25]. Liao et al. engineered *E. coli* to produce various alcohols by overexpressing different heterologous 2-keto-acid decarboxylases (KDCs) and alcohol dehydrogenases (ADHs); 2-PE was detected, and the highest titer of 2-PE (57 mg/L) was obtained when *ARO10* and *ADH2* from *S. cerevisiae* were co-expressed [24]. However, for the recombinant *E. coli* systems harboring the foreign genes, the overexpression of all the genes in soluble and active forms is always a bottleneck [25]. Therefore, developing new enzymes, especially finding highly specific and active phenylpyruvate decarboxylases in prokaryotes, is critical for the biosynthesis of 2-PE. Although 2-PE has been detected in the cultures of several bacterial species, including *Achromobacter eurydice* [26], *Acinetobacter calcoaceticus* [27], *Pseudomonas putida* [28], *Nocardia* sp. 239 [29], and *Thauera aromatica* [30], indicating that de-novo synthesis of 2-PE exists in some bacteria, no further progress has been reported.

We have previously reported the isolation and identification of a new strain, *Enterobacter* sp. CGMCC 5087, which can produce 2-PE using a de novo synthetic pathway with monosaccharide as a carbon source and NH_4_Cl as a nitrogen source [8]. This is the first wild bacterium validated to produce 2-PE using glucose as sole carbon source thus far. However, unlike *E. coli*, this wild strain is not suitable for gene manipulation and fermentation control because of the lack of engineering tools and fermentation control strategies. In addition, this wild strain produces a large amount of acetoin and acetic acid in addition to 2-PE, and these products inhibit the growth of the bacteria. Based on the above reasons, we attempted to search for the 2-PE biosynthetic pathway, specifically, the genes encoding Kdc and Adh in the whole genome of *Enterobacter* sp. CGMCC 5087. Subsequently, the genes of the 2-PE biosynthetic pathway were heterologously overexpressed in *E. coli* BL21(DE3). Then, four upstream pathway genes *aroF/aroG/aroH*, *pheA*, *tktA*, and *ppsA* were screened and overexpressed to construct a highly efficient engineered strain for the production of 2-PE.

## Results

### Genome sequencing, assembly, annotation, and bioinformatic analysis

**Fig. 2:**
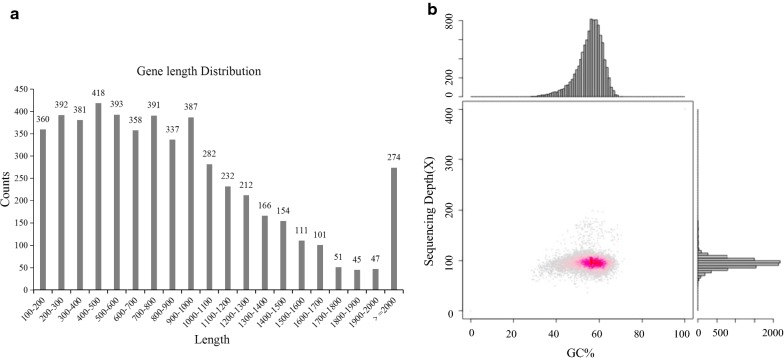
Genome sequencing results for *Enterobacter* sp. CGMCC 5087. **a** Gene length distribution map. **b** Correlation analysis of GC content and depth

In this study, we sequenced the genome of *Enterobacter* sp. CGMCC 5087 using an Illumina HiSeq 2000 sequencing platform (Fig. 2). A total of 504 Mb of data were produced for *Enterobacter* sp. CGMCC 5087 (DDBJ/ENA/GenBank number: QFXN00000000). Based on the assembled result of *Enterobacter* sp. CGMCC 5087 (Fig. 2a), we found that the genome size was 5,110,710 bp; the GC content was 55.56% (Fig. 2b); the number of scaffolds was 56, and the number of contigs was 373. Genome analysis revealed that the *Enterobacter* sp. CGMCC 5087 genome contained 5112 genes; the total length of the genes was 4,505,565 bp, comprising 88.16% of the genome; the number of tRNAs was 57; and the number of rRNAs was 0. All the genes were analyzed by using the KEGG, COG, Swiss-Prot, TrEMBL, NR, and GO databases for functional annotation. By analyzing the genes’ predicted functions, nine genes related to the 2-PE biosynthesis pathway were identified by searching for similar proteins using NCBI (Additional file 2: Table S1).

### Overexpression of candidate Kdc and Adh in *E. coli* BL21 for the detection of 2-PE

**Fig. 3:**
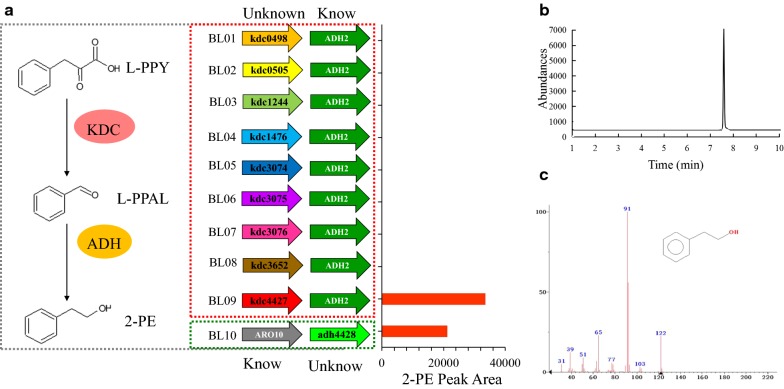
Validation of 2-PE biosynthesis by engineered *E. coli*. **a** Engineered *E. coli* was cultured in LB medium and detected with GC–MS; **b** Graph of GC; **c** graph of GC–MS

In our previous study, *Enterobacter* sp. CGMCC 5087 was validated for the production of 2-PE from phenylpyruvate through the Ehrlich pathway [8]. Therefore, candidate enzyme genes for the 2-PE pathway were predicted from the *Enterobacter* sp. CGMCC 5087 genomic sequence based on their protein sequence homology with known Ehrlich pathway enzymes (Table S1). Next, the predicted candidates *kdc0498*, *kdc0505*, *kdc1244*, *kdc1476*, *kdc3074*, *kdc3075*, *kdc3076*, *kdc3652*, and *kdc4427* from *Enterobacter* sp. CGMCC 5087 were examined in *E. coli* BL21(DE3) in combination with *ADH2* from *S. cerevisiae*. As shown in Fig. 3a, 2-PE was detected in the BL09 strain (*kdc4427* and *ADH2*) by GC–MS, but it was not detected in the others strains; therefore, we concluded that *kdc4427* is the gene encoding phenylpyruvate decarboxylase. Furthermore, 2-PE was detected in the BL11 strain (*ARO10* and *adh4428)* by GC–MS, so we hypothesized that *adh4428* is the gene encoding phenylethanol dehydrogenase.

### Comparison of Kdc4427 with yeast ARO10

**Fig. 4:**
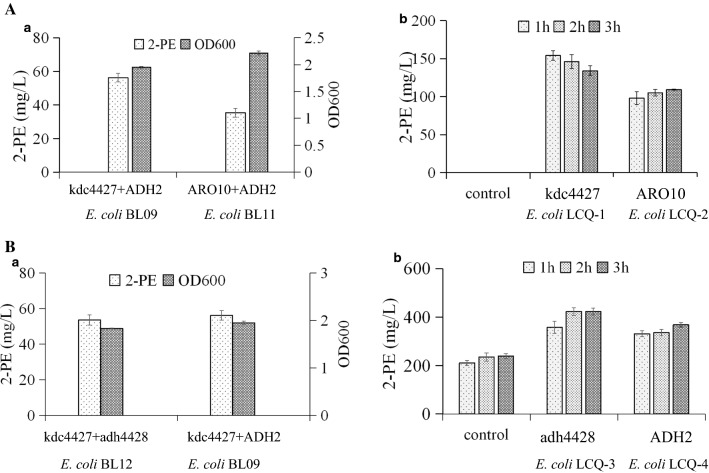
Characterization of Kdc4427 and Adh4428. **A** Characterization of Kdc4427 and comparison with corresponding ARO10. **a** *E. coli* BL09 and *E. coli* BL11 cells were cultivated, and their cell growth (OD_600_) and 2-PE production titers were compared. **b** Phenylpyruvate decarboxylase from Kdc4427 and ARO10 in recombinant *E. coli* BL21(DE3) whole cells. The conversion of 1 g/L phenylpyruvate to 2-PE. **B** Characterization of Adh4428 and comparison with corresponding ADH2. **a** *E. coli* BL12 and *E. coli* BL09 cells were cultivated, and their cell growth (OD_600_) and 2-PE production titers were compared. **b** Phenylpyruvate dehydrogenase from Adh4428 and ADH2 in recombinant *E. coli* BL21(DE3) whole cells. The conversion of 1 g/L phenylacetaldehyde to 2-PE

In previous studies, it was found that ARO10, Pdc5, and Thi3 from *S. cerevisiae*, Kivd from *Lactococcus lactis*, and Pdc from *Clostridium acetobutylicum* can decarboxylate phenylpyruvate, with ARO10 showing the best properties [14, 24, 31]. Here, we compared ARO10 with Kdc4427. As shown in Fig. 4A.a, the expression of *ADH2* with *kdc4427* led to production of 56 mg/L 2-PE in the shake-flask fermentation, which was higher than the production achieved with *ARO10* (35 mg/L). Based on this result, Kdc4427 is more suitable for 2-PE production than ARO10 in the host *E*. *coli* BL21(DE3).

A similar result was also observed in the whole-cell bioconversion. As shown in Fig. 4A.b, *E. coli* LC01 harboring pETDuet-*kdc4427* produced more 2-PE from phenylpyruvate than did LC02 harboring pETDuet-*ARO10*, suggesting that Kdc4427 enzymatic activity is higher than that of ARO10 during 2-PE production with the host *E*. *coli* BL21(DE3). In addition, we found an interesting phenomenon: PAAL was not detected during conversion. One possible reason is that endogenous alcohol dehydrogenase of *E. coli* can catalyze the conversion of all PAAL produced by KDCs to 2-PE. In fact, three candidate genes—*yqhD*, *yjgB*, and *yahK*—have been identified, and *yqhD* has been experimentally confirmed as a broad-substrate alcohol dehydrogenase [32]. In addition, this result suggested that KDCs are rate-limiting enzyme in the biosynthesis of 2-PE with *E*. *coli* BL21(DE3).

To find out the reasons why Kdc4427 is more efficient in the production of 2-PE than ARO10, the protein sequences of them were analyzed. ARO10 has three substrate-bound amino acid residues, which are I335, Q448, and M624, respectively [33]. Three substrate-bound amino acid residues of KDC4427 are predicted to be T290, A387, and I542 by using a homology model. Then comparing the Clustalw base sequences, we found that the substrate-bound sites of ARO10 and Kdc4427 are at the same position in the structure (Additional file 1: Figure S1). And Kdc4427 is also predicted to be an indolepyruvate decarboxylase (IPDC). However, why it has a higher phenylpyruvate decarboxylase activity needs to be further studied.

### Comparison of Adh4428 with yeast ADH2

To better characterize Adh4428, it was compared with the commonly used alcohol dehydrogenase ADH2 from *S. cerevisiae* in the shake-flask fermentation and whole-cell bioconversion. As shown in Fig. 4B.a, the 2-PE yield in *E. coli* BL12 harboring pETDuet-*kdc4427* and pACYCDuet-*adh4428* was approximately the same as that in *E. coli* BL09 harboring pETDuet-*kdc4427* and pACYCDuet-*ADH2*. One possible reason is that alcohol dehydrogenase is not the rate-limiting step in the biosynthesis of 2-PE. Thus, we conducted whole-cell bioconversion for comparison. Cells with no heterologous gene, LC03 cells (pACYCDuet-*adh4428*), and LC04 cells (pACYCDuet-*ADH2*) were collected in 10 mL PBS buffer, and PAAL was added to a final concentration of 1 g/L PAAL. As shown in Fig. 4B.b, the control cells produced approximately 200 mg/L 2-PE. This result confirms the previous hypothesis that endogenous alcohol dehydrogenase of *E. coli* can catalyze the conversion PAAL to 2-PE. Overexpression of Adh4428 in *E. coli* produced approximately 420 mg/L 2-PE, which was slightly higher than that produced by the overexpression of ADH2. The data were further analyzed using SPSS 19.0 (SPSS, Chicago, IL, USA). Independent samples *t*-tests were used to compare the differences in the catalytic efficiency of PPY to 2-PE between Adh4428 and ADH2. *p*-values < 0.05 were considered statistically significant. The results showed that they did not have a significant difference in the catalytic efficiency of PPY to 2-PE in the first hour (*p*-values 0.119), meanwhile they had significant differences in the second and third hours (*p*-values both 0.000). Anyway, Adh4428 was preferred for the 2-PE production compared with ADH2. In addition, we also attempted to express only Kdc4427 in *E. coli* BL21(DE3) to produce 2-PE with relying on endogenous dehydrogenation. The 2-PE yield in *E. coli* LCQ-1 harboring pETDuet-*kdc4427* was lower than that in *E. coli* BL12 harboring pETDuet-*kdc4427* and pACYCDuet-*adh4428* (Additional file 1: Figure S2). Therefore, though overexpression of heterogenous Adh4428 may be a burden to the host, it still necessarily needed for 2-PE production in *E. coli*.

### Carbon flux optimization of l-Phe biosynthesis

**Fig. 5:**
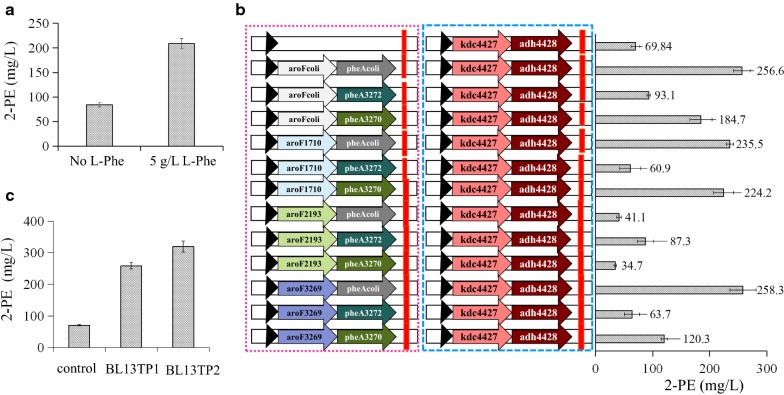
Effects of the overexpression of key upstream genes on 2-PE production. **a** Effect of l-Phe addition on 2-PE production. **b** Effects of the overexpression of candidate *aroF/aroG/aroH* and *pheA* genes on 2-PE production. Engineered *E. coli* cells were cultivated, and 2-PE production titers were compared. **c** Effects of overexpression of the *aroF*, *pheA*, *ppsA*, and *tktA* genes on 2-PE production. Engineered *E. coli* cells were cultivated and 2-PE production titers were compared

When 5 g/L l-Phe was added to the medium, 2-PE production was significantly increased from 70 to 210 mg/L in *E. coli* BL13 (Fig. 5a). This result suggests that the l-Phe supply is a limiting factor inside the cell, and increasing the carbon flow to l-Phe should significantly improve the yield of 2-PE via the de novo pathway.

DAHP synthase, which is the first committed step in general aromatic amino acid synthesis, controls carbon flow into l-Phe biosynthesis. *E. coli* contains three isoenzymes of DAHP synthase occur, encoded by *aroF* (Tyr-sensitive DAHP synthase), *aroG* (Phe-sensitive enzyme), and *aroH* (Tryptophane-sensitive enzyme), respectively [34]. According to known genome sequencing from *E. coli* and gene function prediction, *aroG1710* may encode a Phe-sensitive DAHP synthase; *aroF3269* may encode a Tyr-sensitive enzyme, and *aroH2193* may encode a Tryptophane-sensitive enzyme. In an attempt to further increase 2-PE production by the *E. coli* strain, we examined the combinatorial effects of candidate *aroF/aroG/aroH* and *pheA*. Specifically, *aroG1710*, *aroH2193*, *aroF3269*, *pheA3270, and pheA3272* from *Enterobacter* sp. CGMCC 5087, and *aroFcoli* and *pheAcoli* from *E. coli*, were evaluated for their ability to produce 2-PE. Overexpressing *pheAcoli* together with *aroFcoli* or *aroF3269* generated the highest production of 2-PE among all combinations, with yields of 256 and 258 mg/L, respectively. These yields were higher than that achieved with *aroG1710* (235 mg/L) and are much higher than that achieved with *aroH2193* (41 mg/L). These results suggested Tyr-repressible *aroF* may have the greatest effect on l-Phe biosynthesis among the DAHP synthase genes (*aroF, aroG*, and *aroH*). In addition, the effects of *pheAcoli*, *pheA3272*, and *pheA3270* on 2-PE production were compared. As shown in Fig. 5b, the largest outputs of 2-PE are from the strains harboring *pheAcoli*, followed by the strains harboring *pheA3270*, and then the strains harboring *pheA3272*. In summary, after 24 h of cultivation, the 2-PE titers reached a maximum of approximately 260 mg/L with co-expression of *aroF* and *pheA*, while the control (harboring pACYCDuet-*kdc4427*–*adh4428* and pETDuet-1) produced approximately 70 mg/L 2-PE (Fig. 5c). These results confirmed that the *aroF* and *pheA* genes can help to improve l-Phe and l-Phe derivatives production, similar to the results reported in previous studies [35, 36].

### Carbon flux optimization of carbon central metabolic pathways

According to the phenylpyruvate pathway, two molecules of PEP and one molecule of E4P from carbon central metabolism are required to produce one molecule of l-Phe. PEP is predominantly utilized in the phosphotransferase system (PTS), which is responsible for the translocation and phosphorylation of glucose, converting one PEP molecule to pyruvate (Fig. 1B). Overexpression of *ppsA* encoding PEP synthase can recycle pyruvate generated by PTS-mediated glucose transport to PEP, which is an important approach for increasing the carbon flux from PEP to the l-Phe pathway. Overexpression of *tktA* (which encodes transketolase) is an effective strategy for increasing E4P production [37]. In this study, the *ppsA* and *tktA* genes from *E. coli* were overexpressed in the *E. coli* BL13 (pACYCDuet-*kdc4427*–*adh2*) in order to improve the intracellular pools of PEP and E4P, respectively, and thus enhancing 2-PE production. As shown in Fig. 5c, *E. coli* BL13TP2 (harboring pET-*aroFcoli*–*pheAcoli*–*ppsA*–*tktA* and pACYC-*kdc4427*–*adh4428*) accumulated 320 mg/L 2-PE after 24 h of fermentation, which represents 123% and 457% improvements in 2-PE production by *E. coli* BL13AP1 (harboring pET-*ppsA*–*tktA* and pACYC-*kdc4427*–*adh4428*) and *E. coli* BL13AP0 (harboring pACYCDuet-*kdc4427*–*adh4428*), respectively.

### 2-PE toxicity assay

**Fig. 6:**
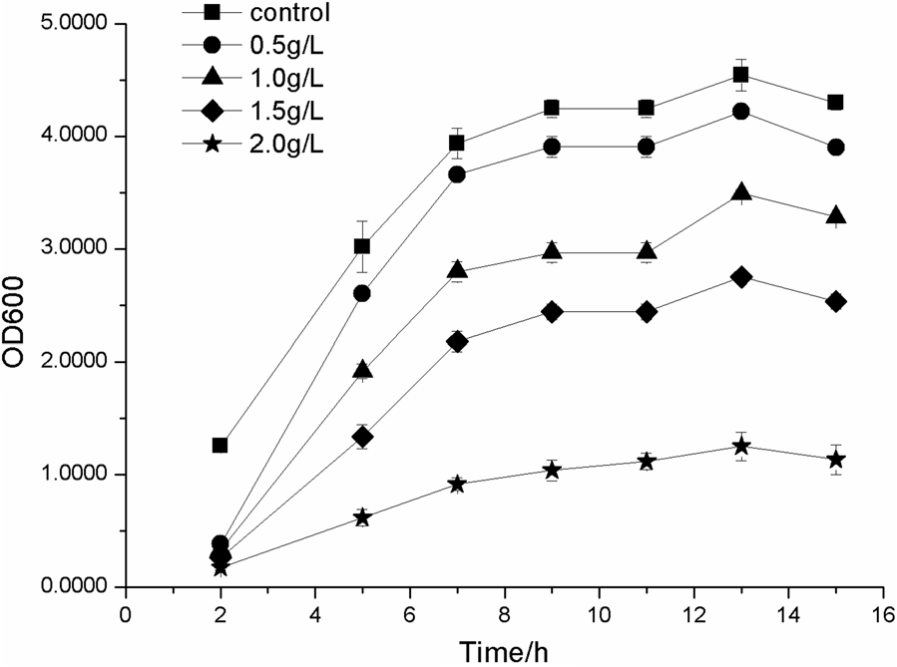
Tolerance of *E. coli* BL21(DE3) cells to 2-PE toxicity. Growth response of *E. coli* BL21(DE3) cells to 0, 0.5 g/L, 1.0 g/L, 1.5 g/L, 2.0 g/L 2-PE in LB medium. Error bars represent one standard deviation from triplicate experiments

To evaluate 2-PE toxicity on *E. coli* BL21(DE3), the effect of exogenous addition of 2-PE at different concentrations (0.5 g/L, 1 g/L, 1.5 g/L, and 2 g/L) on growing cultures was investigated. An OD_600_ of 4.7 was reached in the absence of 2-PE (supplemental material). When the concentration of 2-PE was 0.5 g/L, slight growth inhibition was observed. With increasing concentrations of 2-PE, bacterial cell growth inhibition became more apparent; in particular, when the concentration of 2-PE increased to 2 g/L, bacterial cells were severely inhibited, and the OD_600_ was maintained at approximately 1.0 (Fig. 6). In situ product removal (ISPR) can help to ease the burden of end-product toxicity on bacteria. Various kinds of solvents, such as oleic acid, oleyl alcohol, miglyol, isopropyl myristate, and polypropylene glycol, have been tested for their ability to improve the production of 2-PE compounds [13, 19, 22, 38]. In addition, ionic liquids were also used for ISPR [39] as well as solid phase extraction [40]. In the future, ISPR in combination with genetic approaches could also be used to increase 2-PE tolerance and production capacity of engineered *E. coli*.

## Discussion

PE is a higher aromatic alcohol widely used in the perfumery, cosmetics, and food industries and even in biofuels. In our previous study, a new strain *Enterobacter* sp. CGMCC 5087 was isolated and verified to produce 2-PE using a de novo synthetic pathway with monosaccharide as a carbon source and NH_4_Cl as a nitrogen source [8]. To investigate the prokaryotic 2-PE biosynthesis pathway, the whole genome of *Enterobacter* sp. CGMCC 5087 was sequenced, and we found that overexpression of the *kdc4427* and *adh4428* genes in *E. coli* BL21(DE3) could cause the accumulation of 2-PE. To the best of our knowledge, Kdc4427 is the only phenylpyruvate decarboxylase found and verified in bacteria thus far. To better characterize Kdc4427, ARO10 from *S. cerevisiae* was used for comparison in the shake-flask fermentation and in whole-cell conversion. Based on our results, Kdc4427 has higher phenylpyruvate decarboxylase activity than ARO10. In addition, we also identified and characterized Adh4428, which has higher phenylacetaldehyde dehydrogenase activity than Adh2 from *S. cerevisiae*. Interestingly, we found that endogenous *E. coli* alcohol dehydrogenase can also catalyze the conversion of PAAL to 2-PE. In this study, we describe the engineering of *E. coli* (*kdc4427, adh4428*, *aroF*, *pheA*, *tktA*, and *ppsA*) for 2-PE production from glucose, leading to a final production titer of 320 mg/L (with productivity of 13.3 g/L/h) in shake-flask fermentation, which represents a 4.6-fold improvement over the control strains (*kdc4427* and *adh4428*). To the best of our knowledge, this is the highest titer of de novo production of 2-PE reported in engineered *E. coli* in shake-flask fermentation.

Previously, *E. coli* was also used as a cell factory to product 2-PE. For instance, Liao et al. overexpressed *ARO10* and *ADH2* from *S. cerevisiae* in *E.coli* BW25113/F′ [traD36, proAB+, lacIq ZΔM15], and the resulting strain produced 57 mg/L 2-PE in 40 h [24]. In this study, we also introduced *ARO10* and *ADH2* into *E. coli* and attained 35 mg/L 2-PE in 24 h. Subsequently, Hwang et al. constructed the yeast Ehrlich pathway into *E. coli*, but failure to overexpress all exogenous proteins in a soluble and active form prevented a high yield of 2-PE [18], and they ultimately used l-Phe as feedstock. The discovery of Kdc4427 compensates for the lack of prokaryotic phenylpyruvate decarboxylases and provides new genes for the bioengineering of 2-PE and its derivatives in the future. Recently, Kang et al. attempted to construct a heterologous pathway to produce 2-PE in *E. coli* by overexpressing *ADH1* from *S. cerevisiae*, *KDC* from *Pichia pastoris GS115*, *pheA*^*fbr*^ and *aroF*^*wt*^, and this modified strain ultimately produced 285 mg/L 2-PE in semisynthetic medium containing 0.4 g/L l-Tyr and 3 g/L yeast extract (2% l-Phe). This study also introduced *pheA* and *aroF* into *E. coli* BL13, and the resulting strain produced 260 mg/L 2-PE in a synthetic medium. Although the 2-PE titer was slightly lower than previously reported due to the different compositions of the medium, this study really achieved de novo production of 2-PE from glucose in engineered *E. coli*. Furthermore, after the stain was modified to be *E. coli* BL13TP2 (harboring pET-*aroFcoli*–*pheAcoli*–*ppsA*–*tktA* and pACYC-*KDC4427*–*Adh4428*), the de novo production of 2-PE was achieved as high as 320 mg/L after only 24 h of fermentation.

The maximum theoretical yield coefficients maxY_Phe_/Glc were calculated to be 0.55 g/g based on the known stoichiometry of l-Phe biosynthesis from glucose, in an engineered strain in which either the PTS was inactive or PYR was recycled back to PEP [41, 42]. Moreover, based on the hypothesis that complete conversion of all endogenously produced l-Phe to 2-PE is possible, the maximum theoretical yield (eng. maxY_2-PE_/Glc) was calculated to be 0.41 g/g. According to this value, *E. coli* BL13TP2 (320 mg/L) strains reached yields of 0.053 g/g, corresponding to 12.9% of the eng. maxY_2-PE_/Glc. The above data indicate that it is possible to achieve a higher yield. Major challenges may come from low enzymatic activity and flux imbalance.

Although Kdc4427 has higher enzymatic activity than ARO10, Kdc4427 is still the rate-limiting enzyme in the engineered 2-PE biosynthesis pathway because PAAL titers were observed to remain low or disappear throughout, indicating that almost all of the synthesized PAAL catalyzed by Kdc4427 was quickly converted to 2-PE by the endogenous alcohol dehydrogenase of *E. coli* (Fig. 4A.b). Therefore, methods to improve the activity of KDCs should be considered first. Significant achievements have been made via protein engineering, such as the combination of rational design and directed evolution [43–47]. Metabolic flow imbalance is another problem that needs to be solved to improve 2-PE production. To address this problem, rational strategies to regulate gene expression have been developed, such as the application of inducible promoters, the use of non-native RNA polymerase [46], and replacement of the ribosome binding site [47], as well as multivariate modular metabolic engineering [48]. In addition, biosensors [49, 50], modular scaffold strategies [50], and the compartmentalization of enzymes [51] have been employed to regulate metabolic flux.

## Conclusions

**Fig. 7:**
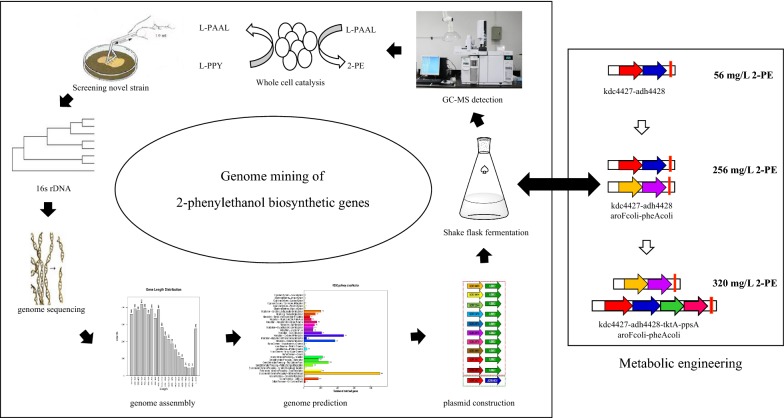
General flow chart of this study

This study obtained a phenylpyruvate decarboxylase (Kdc4427) and phenylacetaldehyde dehydrogenase (Adh4428) from *Enterobacter* sp. *CG*MCC 5087. Next, we introduced *kdc4427* and *adh4428* into *E. coli* BL(DE3), resulting in a 2-PE titer of 56 mg/L from glucose in shake flask cultures, which was higher than that achieved in *E. coli* BL(DE3) harboring *Aro10* and *ADH2* from *S. cerevisiae* (35 mg/L) under the same conditions. Then the upstream shikimate pathways genes *aroF* and *pheA* were overexpressed in the *E. coli* BL13 strain, which led to final 2-PE production at a titer of 258 mg/L in shake-flask fermentation, which representing a 369% improvement in 2-PE production over that achieved in the *E. coli* BL13 strain. Moreover, when we continued to overexpress the central metabolic pathway genes *tktA* and *ppsA*, the engineered *E. coli* BL13TP2 produced 320 mg/L 2-PE (Fig. 7). To the best of our knowledge, this is the highest titer of de novo 2-PE production in engineered *E. coli* from shake-flask cultures.

## Materials and methods

### Strains, media, and reagents

All strains and plasmids used in this study are listed in Table 1. *E. coli* DH5ɑ was used as the host for DNA manipulation. *E. coli* BL21(DE3) was used as the host to express protein and produce 2-PE. Strains were grown routinely in Luria–Bertani (LB) broth (supplemented with suitable amounts of antibiotics if necessary). To evaluate 2-PE production in shake-flask fermentation, strains were grown in a modified M9 medium consisting of the following components: Na_2_HPO_4_, 6 g/L; KH_2_PO_4_ 3 g/L; NaCl, 0.5 g/L; NH_4_Cl, 1 g/L; MgSO_4_·7H_2_O, 0.492 g/L; CaCl_2_, 0.11098 g/L; thiamine–HCl, 0.01 g/L; glucose, 15 g/L and 1 mL/L trace element solution that includes 0.37 g/L (NH_4_)_6_Mo_7_O_24_·4H_2_O, 0.29 g/L ZnSO_4_·7H_2_O, 2.47 g/L H_3_BO_4_, 0.25 g/L CuSO_4_·5H_2_O, and 1.58 g/L MnCl_2_·4H_2_O. DNA polymerase and DNA marker were purchased from TransGen Biotech (Beijing, China). Restriction enzymes and DNA ligase were purchased from Thermo Fisher Scientific (Shanghai, China).

**Table 1:** Plasmids and strains used in this study

Name	Relevant characteristics	References
Plasmids		
pETDuet-1	ColE1(pBR322) ori; Amp^r^; P_T7_	Novagen
pETDuet-*KDC0498*	ColE1(pBR322) ori; Amp^r^; P_T7_-*kdc0498*	This work
pETDuet-*KDC0505*	ColE1(pBR322) ori; Amp^r^; P_T7_-*kdc0505*	This work
pETDuet-*KDC1244*	ColE1(pBR322) ori; Amp^r^; P_T7_-*kdc1244*	This work
pETDuet-*KDC1476*	ColE1(pBR322) ori; Amp^r^; P_T7_-*kdc1476*	This work
pETDuet-*KDC3074*	ColE1(pBR322) ori; Amp^r^; P_T7_-*kdc3074*	This work
pETDuet-*KDC3075*	ColE1(pBR322) ori; Amp^r^; P_T7_-*kdc3075*	This work
pETDuet-*KDC3076*	ColE1(pBR322) ori; Amp^r^; P_T7_-*kdc3076*	This work
pETDuet-*KDC3652*	ColE1(pBR322) ori; Amp^r^; P_T7_-*kdc3652*	This work
pETDuet-*KDC4427*	ColE1(pBR322) ori; Amp^r^; P_T7_-*kdc4427*	
pETDuet-*KDC4472*	ColE1(pBR322) ori; Amp^r^; P_T7_-*kdc4472*	This work
pETDuet-*Aro10*	ColE1(pBR322) ori; Amp^r^; P_T7_-*ARO10*	This work
pETDuet-*aroFcoli*–*pheAcoli*	ColE1(pBR322) ori; Amp^r^; P_T7_-*aroFcoli*–*pheAcoli*	Reference [37]
pETDuet-*aroFcoli*-*pheA3272*	ColE1(pBR322) ori; Amp^r^; P_T7_-*aroFcoli*-*pheA3272*	This work
pETDuet-*aroFcoli*-*pheA3270*	ColE1(pBR322) ori; Amp^r^; P_T7_-*aroFcoli*-*pheA3270*	This work
pETDuet-*aroG1710*-*pheAcoli*	ColE1(pBR322) ori; Amp^r^; P_T7_-*aroG1710*-*pheAcoli*	This work
pETDuet-*aroG1710*-*pheA3272*	ColE1(pBR322) ori; Amp^r^; P_T7_-*aroG1710*-*pheA3272*	This work
pETDuet-*aroG1710*-*pheA3270*	ColE1(pBR322) ori; Amp^r^; P_T7_-*aroG1710*-*pheA3270*	This work
pETDuet-*aroH2193*-*pheAcoli*	ColE1(pBR322) ori; Amp^r^; P_T7_-*aroH2193*-*pheAcoli*	This work
pETDuet-*aroH2193*-*pheA3272*	ColE1(pBR322) ori; Amp^r^; P_T7_-*aroH2193*-*pheA3272*	This work
pETDuet-*aroH2193*-*pheA3270*	ColE1(pBR322) ori; Amp^r^; P_T7_-*aroH2193*-*pheA3270*	This work
pETDuet-*aroF3269*-*pheAcoli*	ColE1(pBR322) ori; Amp^r^; P_T7_-*aroF3269*-*pheAcoli*	This work
pETDuet-*aroF3269*-*pheA3272*	ColE1(pBR322) ori; Amp^r^; P_T7_-*aroF3269*-*pheA3272*	This work
pETDuet-*aroF3269*-*pheA3270*	ColE1(pBR322) ori; Amp^r^; P_T7_-*aroF3269*-*pheA3270*	This work
pTrcHis2B-*AtPAL*-*FDC1*-*ppsA*–*tktA*	pBR322 ori; Amp^r^; P_Trc_-*AtPAL*-*FDC1*-*ppsA*–*tktA*	Reference [37]
pETDuet-*aroFcoli*–*pheAcoli*–*ppsA*–*tktA*	ColE1(pBR322) ori; Amp^r^; P_T7_-*aroFcoli*–*pheAcoli*–*ppsA*–*tktA*	This work
pACYCDuet-1	P15A origin; Cm^R^; P_T7_	Novagen
pACYCduet-*ADH2*	P15A origin; Cm^R^; P_T7_-*ADH2*	This work
pACYCduet-*adh4428*	P15A origin; Cm^R^; P_T7_-*adh4428*	This work
pACYCduet-*kdc4427*–*adh4428*	P15A origin; Cm^R^; P_T7_-*kdc4427*–*adh4428*	This work
Strains		
*E. coli* BL21(DE3)	*E. coli B dcm ompT hsdS*(rB^−^mB^−^) *gal*	Takara
*E. coli* DH5α	*deoR*, recA1, endA1, hsdR17(rk^−^, mk^+^), phoA, supE44, λ^−^, thi^−1^, gyrA96, relA1	Invitrogen
*E. coli* BL01	*E. coli* BL21(DE3) harboring pETDuet-*kdc0428* and pACYCDuet-*ADH2*	This work
*E. coli* BL02	*E. coli* BL21(DE3) harboring pETDuet-*kdc0505* and pACYCDuet-*ADH2*	This work
*E. coli* BL03	*E. coli* BL21(DE3) harboring pETDuet-*kdc1244* and pACYCDuet-*Adh2*	This work
*E. coli* BL04	*E. coli* BL21(DE3) harboring pETDuet-*kdc1476* and pACYCDuet-*ADH2*	This work
*E. coli* BL05	*E. coli* BL21(DE3) harboring pETDuet-*kdc3074* and pACYCDuet-*ADH2*	This work
*E. coli* BL06	*E. coli* BL21(DE3) harboring pETDuet-*kdc3075* and pACYCDuet-*ADH2*	This work
*E. coli* BL07	*E. coli* BL21(DE3) harboring pETDuet-*kdc3076* and pACYCDuet-*Adh2*	This work
*E. coli* BL08	*E. coli* BL21(DE3) harboring pETDuet-*kdc3653* and pACYCDuet-*ADH2*	This work
*E. coli* BL09	*E. coli* BL21(DE3) harboring pETDuet-*kdc4427* and pACYCDuet-*ADH2*	This work
*E. coli* BL10	*E. coli* BL21(DE3) harboring pETDuet-*ARO10* and pACYCDuet-*Adh4428*	This work
*E. coli* BL11	*E. coli* BL21(DE3) harboring pETDuet-*ARO10* and pACYCDuet-*ADH2*	This work
*E. coli* BL12	*E. coli* BL21(DE3) pETDuet-*kdc442*7 and pACYCDuet-a*dh4428*	This work
*E. coli* BL13	*E. coli* BL21(DE3) harboring pACYCDuet-*kdc4427*–*adh4428*	This work
*E. coli* BL13AP1	*E. coli* BL21(DE3) harboring pETDuet-*aroFcoli*–*pheAcoli* and pACYCDuet-*kdc4427*–*adh4428*	This work
*E. coli* BL13AP2	*E. coli* BL21(DE3) harboring pETDuet-*aroFcoli*–*pheA3272* and harboring pACYCDuet-*kdc4427*–*adh4428*	This work
*E. coli* BL13AP3	*E. coli* BL21(DE3) harboring pETDuet-aroFcoli–pheA3270 and pACYCDuet-*kdc*4427–*adh4428*	This work
*E. coli* BL13AP4	*E. coli* BL21(DE3) harboring pETDuet-*aroG1710*-*pheAcoli* and pACYCDuet-*kdc4427*–*adh4428*	This work
*E. coli* BL13AP5	*E. coli* BL21(DE3) harboring pETDuet-*aroG1710*–*pheA3272* and pACYCDuet-*kdc4427*–*adh4428*	This work
*E. coli* BL13AP6	*E. coli* BL21(DE3) harboring pETDuet-*aroG1710*–*pheA3270* and pACYCDuet-*kdc4427*–*adh4428*	This work
*E. coli* BL13AP7	*E. coli* BL21(DE3) harboring pETDuet-*aroH2193*-*pheAcoli* and pACYCDuet-*kdc4427*–*adh4428*	This work
*E. coli* BL13AP8	*E. coli* BL21(DE3) harboring pETDuet-*aroH2193*–*pheA3272* and pACYCDuet-*kdc4427*–*adh4428*	This work
*E. coli* BL13AP9	*E. coli* BL21(DE3) harboring pETDuet-*aroH2193*–*pheA3270* and pACYCDuet-*kdc4427*–*adh4428*	This work
*E. coli* BL13AP10	*E. coli* BL21(DE3) harboring pETDuet-*aroF3269*–*pheAcoli* and pACYCDuet-*kdc4427*–*adh4428*	This work
*E. coli* BL13AP11	*E. coli* BL21(DE3) harboring pETDuet-*aroF3269*–*pheA3272* and pACYCDuet-*kdc4427*–*adh4428*	This work
*E. coli* BL13AP12	*E. coli* BL21(DE3) harboring pETDuet-*aroF3269*–*pheA3270* and pACYCDuet-*kdc4427*–*adh4428*	This work
*E. coli* BL13TP1	*E. coli* BL21(DE3) harboring pETDuet-*tktA*–*ppsA* and pACYCDuet-*kdc4427*–*adh4428*	This work
*E. coli* BL13TP2	*E. coli* BL21(DE3) harboring pETDuet-*tktA*–*ppsA*–*aroFcoli*–*pheAcoli* and pACYCDuet-*kdc4427*–*adh4428*	This work
*E. coli LCQ*-*1*	*E. coli* BL21(DE3) harboring pETDuet-*kdc4427*	This work
*E. coli LCQ*-*2*	*E. coli* BL21(DE3) harboring pETDuet-*ARO10*	This work
*E. coli LCQ*-*3*	*E. coli* BL21(DE3) harboring pETDuet-*adh4428*	This work
*E. coli LCQ*-*4*	*E. coli* BL21(DE3) harboring pETDuet-*ADH2*	This work

### Genome sequencing, genome assembly, and gene prediction

Genomic DNA of *Enterobacter* sp. CGMCC 5087 was extracted utilizing the E.Z.N.A. Bacterial DNA Kit (Omega, Beijing, China). The genome was sequenced using high-throughput Solexa paired-end sequencing technology at the Beijing Genomics Institute (BGI) (Shenzhen, China). Genomic DNA was fragmented randomly, and then DNA fragments of the required length were retained by electrophoresis. After this, we ligated adapters to the DNA fragments and conducted cluster preparation prior to sequencing. Before assembling the fragments, we used k-mer analysis to estimate the size of the genome (the assembled result was the real genome size), the degree of heterozygosity and the degree of duplication. Gene ontology (GO), Kyoto Encyclopedia of Genes and Genomes (KEGG), and Swiss-Port database were used for gene annotation.

### Plasmid construction for 2-PE functional gene identification

The plasmids used in this study are listed in Table 1 and the primers used are listed in Table 2. *Enterobacter* sp. CGMCC 5087 was used as template for cloning candidate *KDC genes*, *the Adh gene, aroF/aroH/aroG genes and pheA genes. Aro10* from *S. cerevisiae* was synthesized by GenScript (Beijing, China). *Adh2* from *S. cerevisiae* was cloned by PCR. *TktA*, *ppsA*, *aroF coli*, and *pheA coli* from *E. coli* BL21(DE3) were cloned by PCR. All constructed plasmids were verified by both colony PCR and Sanger sequencing.

**Table 2:** Primers used in this study

Primers	Nucleotide sequence^a^
pETDuet-*kdc0498*-F	CGCggatcctATGAATCACATGAATAAAC
pETDuet-*kdc0498*-R	CCCaagcttATGCGCTTGTAAGACC
pETDuet-*kdc0505*-F	CGCggatcctGTGGCAAAAGTGGAACCCG
pETDuet-*kdc0505*-R	cgaattcTCATGCCATCCCCTTCGC
pETDuet-*kdc1244*-F	CGCggatcctATGAAACGACTCATTGTTGG
pETDuet-*kdc1244*-R	CCCaagcttCAGGCCCCTTGCCAGCG
pETDuet-*kdc1476*-F	CGCggatcctATGAACACCTTCGACAAAC
pETDuet-*kdc1476*-R	CCCaagcttACAGGTTATCTGGAAAG
pETDuet-*kdc3074*-F	CGCggatcctATGACGGGGGCAACGGGC
pETDuet-*kdc3074*-R	CCCaagcttCAAATGCCATCTTTATTCTC
pETDuet-*kdc3075*-F	CGCggatcctATGGCATTTGATGATTTGAG
pETDuet-*kdc3075*-R	cagagctcTTATTGACGTGCTGCCAGC
pETDuet-*kdc3076*-F	CGCggatcctATGATTTGTCCACGTTGTGC
pETDuet-*kdc3076*-R	CCCaagcttACAGCAGCGGCGGGATTG
pETDuet-*kdc3652*-F	CGCggatcctATGATTAAATCATTAACGTCC
pETDuet-*kdc3652*-R	cgaattcTCAATCTGCGGAAATGGC
pETDuet-*kdc4427*-F	CGCggatcctATGCGTACCCCATACTGC
pETDuet-*kdc4427*-R	ATAAGAATgcggccgcCAGGCGCTATTGCGCGC
pETDuet-*ARO10*-F	CGCggatcctATGGCACCTGTTACAATTG
pETDuet-*ARO10*-R	cagagctCTATTTTTTATTTCTTTTAAGTG
pACYC-*ADH2*-F	catcagatctccatcaccatcatcaccacATGTCTATTCCAGAAACTC
pACYC-*ADH2*-R	cggggtaccTTATTTAGAAGTGTCAAC
pACYC-*adh4428*-F	GAagatctCATGGGTTATCAGCCGGACA
pACYC-*adh4428*-R	CCGctcgagTTATTTTGAGCTGTTCAGGATTG
*pheA3272*-F	GA agatctc ATGACACCGGAAAACCCGTTAC
*pheA3272*-R	CCGctcgag TTAGGCCGGGTCAACCG
*pheA3270*-F	GA agatctc ATGGTTGCTGAATTGACCG
*pheA3270*-R	CCGctcgag TTACTGGCGACTGTCATTTG
*aroG1710*-F	GCggatcctATGAATTATCAGAACGACGATTTACGCAT
*aroG1710*-R	ATAAGAATgcggccgcTTAGCCGCGACGCGCTTTTA
*aroH2193*-F	GCggatcctATGAATAAAACCGATGAACTCCG
*aroH2193*-R	ATAAGAATgcggccgcTTAGAAGCGAGAATCAACCG
*aroF3269*-F	GCg gat cct TTGAGGAAAACAACTATCGCA
*aroF3269*-R	ATAAGAATgcggccgc TTAAGCCAGACGCGTCG
*ppsA*–*tktA*-F	TCCGctcgagTTAAGGAGGTATATATTAATGTCCAACAATGGCTCGT
*ppsA*–*tktA*-R	TCCGcctaggTTACAGCAGTTCTTTTGCTTTCG

### Cultivation conditions and whole-cell conversion conditions

Shake-flask fermentation: A seed culture was prepared by cultivating the strain in 5 mL of LB medium with appropriate antibiotics at 37 °C and 250 rpm overnight. Then, 1 mL of the seed culture was then transferred into a 600-mL salt water bottle containing 100 mL of fermentation medium at 37 °C and 200 rpm. When the cell density reached an OD_600_ of 0.6–0.8, the cultures were induced with 0.4 mM of isopropyl-β-d-thiogalactopyranoside (IPTG), closed with a plug, and then incubated at 30 °C for an additional 24–48 h.

Whole-cell bioconversion: cells were collected after 6 h of culture (resulting in an OD_600_ of ~ 2), centrifuged at 8000×*g* for 5 min, washed with ice-cold PBS buffer (pH = 7.0) twice, and suspended in 10 mL PBS buffer. Finally, the appropriate substrate, PPA or PAAL, was added to the suspension at a final concentration of 1 g/L. Each suspension was then shaken at 32 °C for a total of 3 h. Samples (1 mL) were taken every hour and centrifuged, and 750 mL of supernatant was collected and filtered through 0.22-μm polyether sulfone membranes for HPLC analysis to monitor the production of either 2-PE or PAAL.

### Quantification of 2-PE

After fermentation, the cultures were centrifuged at 8000×*g* for 1 min, and 50 ml of supernatant was extracted with 10 mL of n-heptane. Then, the extract was filtered through 0.22-μm nylon membranes and analyzed by GC–MS to qualify the biosynthesis of 2-PE. M9 medium left uninoculated and treated with the same procedures was used as the control. GC–MS analysis used a previously described procedure [8]. 2-PE production was quantified by GC, with following program: 50 °C for 1 min, which was increased at 20 °C/min to 240 °C and held for 1 min.

The concentrations of PAAL and 2-PE were measured by an HPLC (Waters 1525 series, USA) system with a 250 × 4.6 mm Bio-Rad column (California, USA), a standard 2707 autosampler (Waters, USA) and a Waters 2998 photodiode array detector (PAD) (Waters, USA). Analysis was performed at 30 °C with a mobile phase comprising 70% acetonitrile in water at a flow rate of 0.35 mL/min, and analytes were detected at OD_210nm_.

### 2-PE toxicity assay

A total of 1 mL of seed culture was transferred into a 100-mL triangular flask containing 50 mL LB medium and 2-PE at different concentrations (0, 0.5 g/L, 1.0 g/L, 1.5 g/L, or 2 g/L, respectively). The flasks were incubated at 30 °C and 200 rpm. Cell growth was determined by OD_600_ measurements using a UV/Vis spectrophotometer.

## Additional files

**Additional file 1: Figure S1.** Protein sequences alignment of the Kdc4427 and ARO10 (ClustalX2). **Figure S2.** *E. coli* LCQ-1 and *E. coli* BL12 cells were cultivated, and 2-PE production titers were compared.

**Additional file 2: Table S1.** Candidate genes and their function prediction in this study.
