# Supplementary material for: Genome mining of 2-phenylethanol biosynthetic genes from Enterobacter sp. CGMCC 5087 and heterologous overproduction in Escherichia coli
Source: Biotechnol Biofuels. 2018 Nov 8;11:305. doi: 10.1186/s13068-018-1297-3 (PMC6223000; doi:10.1186/s13068-018-1297-3)

S1

```
KDC4427  1    MRTPYCV---------------------ADYLLDRLTDCGADHLFGVPGDYNLQFLDHVI  39
ARO10    1    M-APVTIEKFVNQEERHLVSNRSATIPFGEYIFKRLLSIDTKSVFGVPGDFNLSLLEYLY  59

KDC4427  40   D----SPDICWVGCANELNASYAADGYARCKG-FAALLTTFGVGELSAMNGIAGSYAEHV  94
ARO10    60   SPSVESAGLRWVGTCNELNAAYAADGYSRYSNKIGCLITTYGVGELSALNGIAGSFAENV  119

KDC4427  95   PVLHIVG--------APGTASQQRGELLHHT--LGDGEFRH-----FYHMSEPITVAQAI  139
ARO10    120  KVLHIVGVAKSIDSRSSNFSDRN---LHHLVPQLHDSNFKGPNHKVYHDMVKDRVACSVA  176

KDC4427  140  LTE--QNACYEIDRVLTTMLRERRPGYLMLPADVAKKAATPPVNALTLRHAHADSAC---  194
ARO10    177  YLEDIETACDQVDNVIRDIYKYSKPGYIFVPADFADMSVTCDNLVNVPRISQQDCIVYPS  236

KDC4427  194  ---LKAFRDAAENRLAMSKRTALLADFLVLRHGLKHALQKWVKDVPMAHATMLMGKGIFD  251
ARO10    237  ENQLSDIINKITSWIYSSKTPAILGDVLTDRYGVSNFLNKLICKTGIWNFSTVMGKSVID  296

KDC4427  252  ERHVGFYGTYSGSASAGAVKEAIEGADTVLCIGTRFTDTLTAGFTHQLTP-SQTIEVQPH  310
ARO10    297  ESNPTYMGQYNGKEGLKQVYEHFELCDLVLHFGVDINEINNGHYTFTYKPNAKIIQFHPN  356

KDC4427  311  ASRVGDVWFTGIPMLQAIE---TLVELCK---------QHVHDTPVPSSQSAMVYPQPDG  358
ARO10    357  YIRLVDTRQGNEQMFKGINFAPILKELYKRIDVSKLSLQYDSNVTQYTNETMRLEDPTNG  416

KDC4427  359  S---LTQDNFWKTLQTFIRPGDIILADQGTSAFGAIDLRLPADVNFIVQPLWGSIGYTLA  415
ARO10    417  QSSIITQVHLQKTMPKFLNPGDVVVCETGSFQFSVRDFAFPSQLKYISQGFFLSIGMALP  476

KDC4427  416  AAFGAQTACPNR----------------RVIVLTGDGAAQLTIQELGSMLRDKQHPIILV  459
ARO10    477  AALGVGIAMQDHSNAHINGGNVKEDYKPRLILFEGDGAAQMTIQELSTILKCNIPLEVII  536

KDC4427  460  LNNEGYTVERAIHGPEQRYNDIALWNWTQIPQAL---------SLDPQAQCWRVSEAEQL  510
ARO10    537  WNNNGYTIERAIMGPTRSYNDVMSWKWTKLFEAFGDFDGKYTNSTLIQCPSKLALKLEEL  596

KDC4427  511  ADVLEKVAHHERLSLIEVMLPKADIPPLLGAITKALEA-RNSA  552
ARO10    597  KNSNKRSG----IELLEVKLGELDFPEQLKCMVEAAALKRNKK  635
```

S2

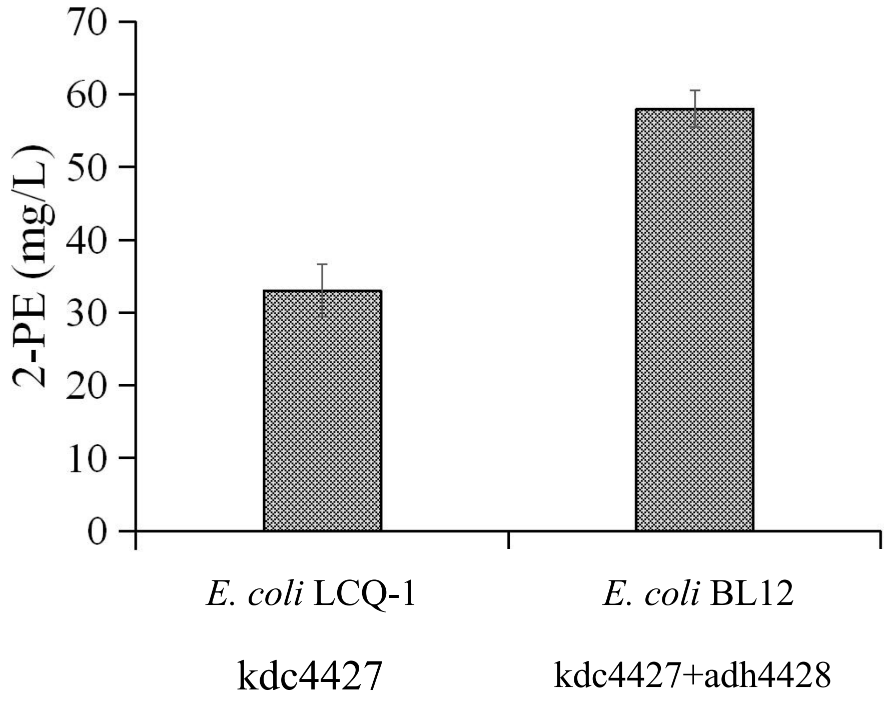

Supplement: Supplementary file 1 — Additional file 1: Figure S1. Protein sequences alignment of the Kdc4427 and ARO10 (ClustalX2). Figure S2. E. coli LCQ-1 and E. coli BL12 cells were cultivated, and 2-PE production titers were compared. [file 13068_2018_1297_MOESM1_ESM.pdf]
