# Supplementary material for: Genome mining of 2-phenylethanol biosynthetic genes from Enterobacter sp. CGMCC 5087 and heterologous overproduction in Escherichia coli
Source: Biotechnol Biofuels. 2018 Nov 8;11:305. doi: 10.1186/s13068-018-1297-3 (PMC6223000; doi:10.1186/s13068-018-1297-3)
Supplement: Supplementary file 2 — Additional file 2: Table S1. Candidate genes and their function prediction in this study. [file 13068_2018_1297_MOESM2_ESM.doc]

**Additional file 2**

**Table S1**

Candidate genes and their function prediction in this study

| Gene location | Gene name in this study | Gene length/product(bp/aa) | Similar polypeptide | I% Identity | organism | Accession no. |
| --- | --- | --- | --- | --- | --- | --- |
| GL000498 | kdc0498 | 927/308 | phenylacetic acid degradation protein | 99 | [*Enterobacter sp*. EGD-HP1] | [KFA85778.1](https://www.ncbi.nlm.nih.gov/protein/667747492?report=genbank&log$=protalign&blast_rank=6&RID=JX0M4TTF011) |
| phenylacetic acid degradation operon negative regulatory protein PaaX | 99 | [*Enterobacter cloacae*] | [WP_062935618.1](https://www.ncbi.nlm.nih.gov/protein/1016091947?report=genbank&log$=protalign&blast_rank=3&RID=JX0M4TTF011) |
| PaaX family transcriptional regulator | 97 | [*Enterobacter cloacae*] | [CZV79616.1](https://www.ncbi.nlm.nih.gov/protein/1014983938?report=genbank&log$=protalign&blast_rank=6&RID=JX0M4TTF011) |
| GL000505 | kdc0505 | 798/265 | phenylacetic acid degradation protein | 99 | [*Enterobacter asburiae*] | [WP_029741868.1](https://www.ncbi.nlm.nih.gov/protein/658549462?report=genbank&log$=protalign&blast_rank=4&RID=JWWH3V07011) |
| ring 1,2-phenylacetyl-CoA epoxidase, NAD(P)H oxidoreductase component | 99 | [*Escherichia coli str*. K-12 substr. MG1655] | [NP_415910.1](https://www.ncbi.nlm.nih.gov/protein/16129353?report=genbank&log$=protalign&blast_rank=1&RID=JWWH3V07011) |
| ferredoxin | 97 | [*Enterobacter sp*. EGD-HP1] | [KFA85750.1](https://www.ncbi.nlm.nih.gov/protein/667747464?report=genbank&log$=protalign&blast_rank=6&RID=JWWH3V07011) |
| 3-ketosteroid-9-alpha-hydroxylase reductase subunit | 33 | [*Mycobacterium tuberculosis* H37Rv] | [NP_218088.1](https://www.ncbi.nlm.nih.gov/protein/15610707?report=genbank&log$=protalign&blast_rank=6&RID=JWWH3V07011) |
| GL001244 | kdc1244 | 570/189 | 3-octaprenyl-4-hydroxybenzoate carboxy-lyase | 89% | [*Escherichia coli str*. K-12 substr. MG1655] | [NP_416814.1](https://www.ncbi.nlm.nih.gov/protein/16130246?report=genbank&log$=prottop&blast_rank=1&RID=JWM1ZXK7011) |
| [aromatic acid decarboxylase](https://blast.ncbi.nlm.nih.gov/smartblast/smartBlast.cgi#alnHdr_15677775) | 49 | [*Neisseria meningitidis* MC58] | [NP_274939.1](https://www.ncbi.nlm.nih.gov/protein/15677775?report=genbank&log$=prottop&blast_rank=2&RID=JWM1ZXK7011) |
| [phenylacrylic acid decarboxylase PAD1](https://blast.ncbi.nlm.nih.gov/smartblast/smartBlast.cgi#alnHdr_398366689) | 50 | [*Saccharomyces cerevisiae* S288c] | [NP_010827.3](https://www.ncbi.nlm.nih.gov/protein/398366689?report=genbank&log$=prottop&blast_rank=3&RID=JWM1ZXK7011) |
| GL001476 | kdc1476 | 504/167 | MULTISPECIES:[phenolic acid decarboxylase](https://blast.ncbi.nlm.nih.gov/smartblast/smartBlast.cgi#alnHdr_654547016) | 100 | [[](https://blast.ncbi.nlm.nih.gov/smartblast/smartBlast.cgi#alnHdr_654547016)*Enterobacter*] | [WP_028014722.1](https://www.ncbi.nlm.nih.gov/protein/654547016?report=genbank&log$=protalign&blast_rank=1&RID=JWS9JH0Z011) |
| [phenolic acid decarboxylase](https://blast.ncbi.nlm.nih.gov/smartblast/smartBlast.cgi#alnHdr_1022651118) | 99 | [*Enterobacter asburiae*] | WP_063409089.1 |
| GL003074 | kdc3074 | 194 | phenolic acid decarboxylase subunit B | 99 | [*Enterobacter sp*. EGD-HP1] | [KFA84695.1](https://www.ncbi.nlm.nih.gov/protein/667746399?report=genbank&log$=protalign&blast_rank=1&RID=JWTSCFRH011) |
| 3-octaprenyl-4-hydroxybenzoate carboxy-lyase UbiX | 100 | [*Enterobacter*] | [WP_023308983.1](https://www.ncbi.nlm.nih.gov/protein/556424056?report=genbank&log$=protalign&blast_rank=4&RID=JWTSCFRH011) |
| aromatic acid decarboxylase | 49 | [*Neisseria meningitidis* MC58] | [NP_274939.1](https://www.ncbi.nlm.nih.gov/protein/15677775?report=genbank&log$=protalign&blast_rank=1&RID=JWTSCFRH011) |
| GL003075 | kdc3075 | 1428/475 | phenolic acid decarboxylase | 99 | [*Enterobacter cloacae*] | [WP_063144742.1](https://www.ncbi.nlm.nih.gov/protein/1016943218?report=genbank&log$=protalign&blast_rank=6&RID=JWVSVY2V011) |
| 4-hydroxybenzoate decarboxylase | 99 | [*Enterobacter cloacae*] | [BAE97712.1](https://www.ncbi.nlm.nih.gov/protein/110331749?report=genbank&log$=protalign&blast_rank=6&RID=JWVSVY2V011) |
| UbiD family decarboxylase | 99 | [*Enterobacter sp*. NFIX59] | [SFI19750.1](https://www.ncbi.nlm.nih.gov/protein/1097978664?report=genbank&log$=protalign&blast_rank=6&RID=JWVSVY2V011) |
| protein vdcC | 99 | [*Enterobacter cloacae* complex] | [WP_023294439.1](https://www.ncbi.nlm.nih.gov/protein/556311838?report=genbank&log$=protalign&blast_rank=4&RID=JWVSVY2V011) |
| GL003076 | kdc3076 | 222/73 | 4-hydroxybenzoate decarboxylase | 99 | [*Enterobacter asburiae*] | [AMX08517.1](https://www.ncbi.nlm.nih.gov/protein/1016277938?report=genbank&log$=protalign&blast_rank=6&RID=JWUWMJY6011) |
| Phenolic acid decarboxylase subunit D |  | *Bacillus subtilis* (strain 168) |  |
| GL003652 | kdc3652 | 563 | acetolactate synthase | 99 | [*Enterobacter asburiae*] | [WP_047648464.1](https://www.ncbi.nlm.nih.gov/protein/835750128?report=genbank&log$=protalign&blast_rank=6&RID=JX2186X2011) |
| benzoylformate decarboxylase | 93 | [*Enterobacter sp*. NFIX59] | [SFI02081.1](https://www.ncbi.nlm.nih.gov/protein/1097980609?report=genbank&log$=protalign&blast_rank=6&RID=JX2186X2011) |
| thiamine pyrophosphate enzyme | 26 | [*Sulfolobus acidocaldarius*] | [WP_011277991.1](https://www.ncbi.nlm.nih.gov/protein/499597257?report=genbank&log$=protalign&blast_rank=6&RID=JX2186X2011) |
| GL004427 | kdc4427 | 552 | indolepyruvate decarboxylase | 99 | [*Enterobacter cloacae*] | [WP_048977829.1](https://www.ncbi.nlm.nih.gov/protein/895869543?report=genbank&log$=protalign&blast_rank=6&RID=JWYWZF2Z011) |
| pyruvate decarboxylase (predicted) | 33 | [*Schizosaccharomyces pombe* 972h-] | [NP_594083.1](https://www.ncbi.nlm.nih.gov/protein/19114995?report=genbank&log$=protalign&blast_rank=6&RID=JWYWZF2Z011) |
| branched-chain-2-oxoacid decarboxylase THI3 | 33 | [Saccharomyces cerevisiae S288C] | [NP_010203.1](https://www.ncbi.nlm.nih.gov/protein/6320123?report=genbank&log$=protalign&blast_rank=7&RID=JWYWZF2Z011) |
| phenylpyruvate decarboxylase ARO10 | 31 | [*Saccharomyces cerevisiae* S288C] | [NP_010668.3](https://www.ncbi.nlm.nih.gov/protein/398366545?report=genbank&log$=protalign&blast_rank=9&RID=JWYWZF2Z011) |
| acetolactate synthase 3 large subunit | 23 | [*Escherichia coli str*. K-12 substr. MG1655] | [YP_025294.2](https://www.ncbi.nlm.nih.gov/protein/90111084?report=genbank&log$=protalign&blast_rank=6&RID=JWYWZF2Z011) |
| GL004428 | kdc4428 | 999/332 | L-glyceraldehyde 3-phosphate reductase | 99 | [*Enterobacter cloacae*] | [WP_048977827.1](https://www.ncbi.nlm.nih.gov/protein/895869540?report=genbank&log$=protalign&blast_rank=6&RID=JXR1AF41011) |
| aldo/keto reductase | 94 | [*Enterobacter sp*. MGH 24] | [WP_023332938.1](https://www.ncbi.nlm.nih.gov/protein/556482651?report=genbank&log$=protalign&blast_rank=4&RID=JXR1AF41011) |
| MULTISPECIES: NADP-dependent oxidoreductase domain containing protein | 93 | [*Enterobacter cloacae* complex] | [WP_020883099.1](https://www.ncbi.nlm.nih.gov/protein/527036357?report=genbank&log$=protalign&blast_rank=4&RID=JXR1AF41011) |
| GL001710 | aroG1710 | 1053/350 | 3-deoxy-7-phosphoheptulonate synthase | 99 | [*Enterobacter cloacae*] | [WP_063143140.1](https://www.ncbi.nlm.nih.gov/protein/1016941612?report=genbank&log$=protalign&blast_rank=6&RID=JYYMCZ0X011) |
| 3-deoxy-D-arabino-heptulosonate-7-phosphate synthase, phenylalanine repressible | 94 | [*Escherichia coli str*. K-12 substr. MG1655] | [NP_415275.1](https://www.ncbi.nlm.nih.gov/protein/16128722?report=genbank&log$=protalign&blast_rank=1&RID=JYYMCZ0X011) |
| phospho-2-dehydro-3-deoxyheptonate aldolase | 64 | [*Neisseria meningitidis* MC58] | [NP_273357.1](https://www.ncbi.nlm.nih.gov/protein/15676225?report=genbank&log$=protalign&blast_rank=1&RID=JYYMCZ0X011) |
| GL002193 | aroH2193 | 1047/328 | 3-deoxy-7-phosphoheptulonate synthase | 99 | [*Enterobacter cloacae*] | [WP_062935557.1](https://www.ncbi.nlm.nih.gov/protein/1016091886?report=genbank&log$=protalign&blast_rank=6&RID=K55KRJBG011) |
| 3-deoxy-D-arabino-heptulosonate-7-phosphate synthase, tryptophan repressible | 88 | [*Escherichia coli str*. K-12 substr. MG1655] | [NP_416219.1](https://www.ncbi.nlm.nih.gov/protein/16129660?report=genbank&log$=protalign&blast_rank=1&RID=K55KRJBG011) |
| phospho-2-dehydro-3-deoxyheptonate aldolase |  | [*Pseudomonas aeruginosa* PAO1] | [NP_250441.1](https://www.ncbi.nlm.nih.gov/protein/15596947?report=genbank&log$=protalign&blast_rank=6&RID=K55KRJBG011) |
| GL003269 | aroF3269 | 1071/356 | 3-deoxy-7-phosphoheptulonate synthase | 99 | [*Enterobacter cloacae*] | [WP_063930426.1](https://www.ncbi.nlm.nih.gov/protein/1028184750?report=genbank&log$=protalign&blast_rank=6&RID=K56A3K8A011) |
| 3-deoxy-D-arabino-heptulosonate-7-phosphate synthase, tyrosine-repressible | 93 | [*Escherichia coli str*. K-12 substr. MG1655] | [NP_417092.1](https://www.ncbi.nlm.nih.gov/protein/16130522?report=genbank&log$=protalign&blast_rank=1&RID=K56A3K8A011) |
| phospho-2-dehydro-3-deoxyheptonate aldolase | 57 | [*Pseudomonas aeruginosa* PAO1] | [NP_250441.1](https://www.ncbi.nlm.nih.gov/protein/15596947?report=genbank&log$=protalign&blast_rank=1&RID=K56A3K8A011) |
| GL003270 | pheA3270 | 1122/373 | bifunctional chorismate mutase/prephenate dehydrogenase | 99 | [*Enterobacter cloacae*] | [WP_048226728.1](https://www.ncbi.nlm.nih.gov/protein/851939715?report=genbank&log$=protalign&blast_rank=6&RID=K56U2F67011) |
| fused chorismate mutase T/prephenate dehydrogenase | 92 | [*Escherichia coli str*. K-12 substr. MG1655] | [NP_417091.1](https://www.ncbi.nlm.nih.gov/protein/16130521?report=genbank&log$=protalign&blast_rank=1&RID=K56U2F67011) |
| GL003272 | pheA3272 | 1161/386 | bifunctional chorismate mutase/prephenate dehydratase | 99 | [*Enterobacter cloacae*] | [WP_063144128.1](https://www.ncbi.nlm.nih.gov/protein/1016942600?report=genbank&log$=protalign&blast_rank=6&RID=K57WB7TM011) |
| chorismate mutase and prephenate dehydratase, P-protein | 95 | [*Escherichia coli str*. K-12 substr. MG1655] | [NP_417090.1](https://www.ncbi.nlm.nih.gov/protein/16130520?report=genbank&log$=protalign&blast_rank=1&RID=K57WB7TM011) |
| multifunctional chorismate mutase P/prephenate dehydratase/DAHP synthetase PheA | 55 | [*Shewanella oneidensis* MR-1] | [NP_716987.1](https://www.ncbi.nlm.nih.gov/protein/24372945?report=genbank&log$=protalign&blast_rank=2&RID=K57WB7TM011) |
